# Dipeptidyl Peptidase-4 Inhibition May Stimulate Progression of Carcinoid Tumor

**DOI:** 10.1155/2015/952019

**Published:** 2015-07-28

**Authors:** Vladimir Pech, Khalid Abusaada, Carlos Alemany

**Affiliations:** ^1^Internal Medicine Residency Program, Florida Hospital, 2501 N. Orange Avenue, Suite 235, Orlando, FL 32804, USA; ^2^Cancer Institute of Florida, Florida Hospital, 2501 N. Orange Avenue, Suite 235, Orlando, FL 32804, USA

## Abstract

Dipeptidyl peptidase-4 (DPP-4) inhibitors, such as saxagliptin, have gained a rapid growth in use in the treatment of type 2 diabetes mellitus in the past decade. Although they are considered to have a good safety profile, controversy exists regarding their potential to stimulate neoplasm growth. We report here a patient with metastatic carcinoid tumor. His disease was stable for several years with plasma serotonin level (which was used to monitor disease progression) in 700–800 ng/mL range. After initiation of treatment with saxagliptin, however, his serotonin level almost doubled (1358 ng/mL), concerning progression of the disease. After discontinuation of saxagliptin, serotonin level returned to baseline quickly, while other laboratory markers, such as complete blood count (CBC), comprehensive metabolic profile (CMP) with liver function tests (LFTs), and lactate dehydrogenase (LD), remained unchanged before, during, and after the treatment with saxagliptin. This temporal correlation suggests a possible interaction between the activity of carcinoid tumors and the use of DPP-4 inhibitors. Although we were not able to find any literature providing a direct evidence that saxagliptin alters progression of the carcinoid tumors, we recommend alternative management for the treatment of diabetes in patients with carcinoid or other neuroendocrine tumors.

## 1. Introduction

Carcinoid tumors are relatively rare neuroendocrine tumors (NET) arising from the lungs and gastrointestinal tract. Their annual incidence in the United States is about 3.65 per 100,000 people [1]. The histological characteristics and clinical behavior of neuroendocrine tumors range from well-differentiated tumors with a relatively benign clinical course to poorly differentiated neuroendocrine carcinomas that resemble small cell or large cell neuroendocrine carcinoma of the lung [2]. Although the term carcinoid is usually reserved for well-differentiated neuroendocrine tumors with more indolent clinical course, they do have the potential to metastasize. Carcinoids are hormonally active tumors. The extent to which carcinoid tumors are regulated by other hormones is not clear; however, they do express receptors for IGF-1, somatostatin, and gastrin overproduction was linked to the development of at least a subtype of carcinoid tumors [3]. Treatment of metastatic carcinoid tumors that are not amenable to resection focuses on control of the symptoms of hormone hypersecretion (the serotonin syndrome) and control of the tumor growth. Most patients are managed with somatostatin analogs, such as octreotide or lanreotide, interferon alfa, and/or the molecularly targeted agents* everolimus* and* sunitinib* that have been shown to improve progression-free survival in patients with metastases from nonfunctioning pancreatic NET [4, 5].

## 2. Case Presentation

**Figure 1:**
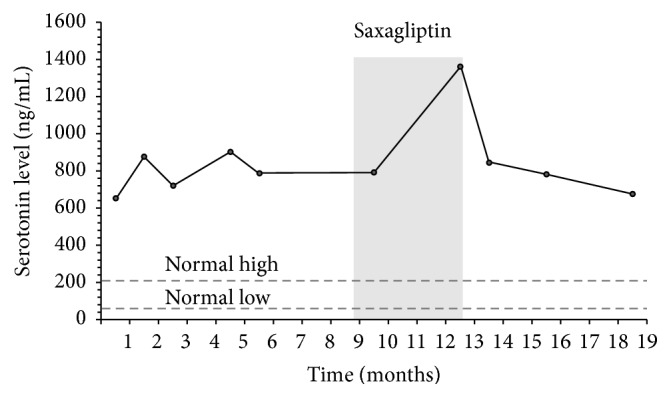
Serotonin level profile (axis *Y*) over period of 19 months. Serotonin level has been stable for several years (last 9 months before initiation of saxagliptin is shown in the figure). 3.5 months after initiation of saxagliptin, serotonin level almost doubled but returned back to baseline within 4 weeks after discontinuation of the treatment. Solid black line represents the patient's serotonin level. Horizontal dashed gray lines represent normal serotonin serum concentrations. Grayed area indicates the frame of saxagliptin exposure.

We report here a 66-year-old Caucasian male patient with recurrent metastatic carcinoid tumor arising from the superior border of the third portion of the duodenum and/or inferior aspect of the pancreatic head with metastatic involvement of the mediastinal lymph nodes. His last surgical resection was 7 years ago. Although the recurrent tumor was unresectable due to anatomical location, his disease has been stable and has been well controlled by medical management with octreotide and later everolimus, without any evidence of progression both clinically and radiologically, and by laboratory for several years. The plasma serotonin level (which was used to monitor disease progression) remained stable in 700–800 ng/mL range. On a recent routine followup, however, his serotonin level almost doubled (1358 ng/mL), concerning progression of the disease. Review of medication revealed a DPP-4 inhibitor, saxagliptin, which was started 3.5 months before this clinical followup for the management of type 2 diabetes mellitus. Treatment with saxagliptin was stopped immediately at that time. Within 4 weeks after discontinuation of saxagliptin, serotonin returned to baseline level (Figure 1), while other laboratory markers, such as complete blood count (CBC), comprehensive metabolic profile (CMP) with liver function tests (LFTs), and lactate dehydrogenase (LD), remained unchanged before, during, and after the treatment with saxagliptin. In addition, we were not able to identify any changes in diet that might have resulted in altered ingestion of tryptophan/serotonin-rich foods. This temporal correlation suggests a possible interaction between the activity of carcinoid tumors and the use of DPP-4 inhibitors. Alternatively, saxagliptin might interfere with degradation of serotonin. Although we were not able to find any literature providing a direct evidence that saxagliptin alters progression of the carcinoid tumors, we recommended alternative management for the treatment of diabetes.

## 3. Discussion

Saxagliptin is a dipeptidyl peptidase-4 inhibitor. DPP-4 inhibitors inhibit activity of dipeptidyl peptidase 4, an enzyme that is responsible for rapid inactivation of glucagon-like peptide 1 (GLP-1). GLP-1 is a native incretin that regulates secretion of insulin and other pancreatic enzymes as well as activity of other extrapancreatic tissues. Because GLP-1 analogues and DPP-4 inhibitors appear to offer many advantages for treating type 2 diabetes, they have gained a rapid growth in use in the past decade. Some preclinical animal studies suggested, however, that GLP-1 analogues and DPP-4 inhibitors may lead to proliferation of pancreatic tissue, including islet *β*-cells, which raised concerns that they could potentially contribute to the development of pancreatitis and increase the risk of pancreatic cancer. Nevertheless, these concerns were not substantiated in follow-up clinical studies and postmarketing surveillance and incretin-based therapies are generally considered to have a good safety profile [6, 7]. On the other hand, the proliferative capacity of these agents remains controversial and recent data indicate their involvement in other extrapancreatic neoplasms. An increased incidence of medullary thyroid cancer was described in some species. DPP-4 was implicated in the development of thyroid neoplasia, such as papillary and follicular cancer [8], and associated with decreased survival of patient with clear cell renal carcinoma [9]. Interestingly, data are emerging suggesting beneficial effects of GLP-1 on colon and breast cancer (reviewed in [10]). With specific regard to the progression of carcinoid tumor and the use of DPP-4 inhibitors, we were not able to identify any reports in the literature.

To the best of the authors' knowledge, this is the first report of possible interaction between incretin-based antidiabetic medications and carcinoid tumor. Although we do not prove a causal relationship between carcinoid tumor progression and the use of saxagliptin, the hormone sensitivity of the carcinoid tumors and the plethora of incretin effects make this interaction highly plausible. Nevertheless, more observations are needed to support this hypothesis. As GLP-1 analogues remain contraindicated in patients with medullary thyroid cancer and in patients with history of multiple endocrine neoplasia syndrome type 2, caution should be used with the use of DPP-4 inhibitors in the management of diabetes in patients with a known history of neoplasm, considering the similar mechanism of action of DPP-4 inhibitors and GLP-1 analogues and the relation of carcinoid tumors to the other NET.
